# Is the Pelvis-Thorax Coordination a Valuable Outcome Instrument to Assess Patients With Hip Osteoarthritis?

**DOI:** 10.3389/fbioe.2019.00457

**Published:** 2020-01-23

**Authors:** Florent Moissenet, Alexandre Naaim, Paul Ornetti, Abderrahmane Bourredjem, Christine Binquet, Claire Morisset, Anais Gouteron, Jean-Francis Maillefert, Davy Laroche

**Affiliations:** ^1^Kinesiology Laboratory, University of Geneva, Geneva, Switzerland; ^2^Université de Lyon, Université Claude Bernard Lyon 1, IFSTTAR, LBMC UMR T9406, Lyon, France; ^3^Centre Hospitalier Universitaire Dijon-Bourgogne, Service de Rhumatologie, Dijon, France; ^4^INSERM, CIC 1432, Module Plurithematique, Plateforme d'Investigation Technologique, Dijon, France; CHU Dijon-Bourgogne, Centre d'Investigation Clinique, Module Plurithématique, Plateforme d'Investigation Technologique, Dijon, France; ^5^INSERM UMR 1093, Cognition, Action et Plasticité sensorimotrice, Dijon; Université de Bourgogne Franche Comté, Dijon, France; ^6^INSERM, CIC 1432, Module Epidémiologie Clinique, Dijon, France; CHU Dijon-Bourgogne, Centre d'Investigation Clinique, Module Epidémiologie clinique/essais cliniques, Dijon, France; ^7^Centre Hospitalier Universitaire Dijon-Bourgogne, service de médecine physique et réadaptation, Dijon, France

**Keywords:** clinical gait analysis, biomechanics, hip, osteoarthritis, outcomes measures, coordination, walking

## Abstract

**Objective:** The evaluation of the disease severity in hip osteoarthritis (OA) patients being currently based on subjective instruments. It would be of interest to develop more objective instruments, for example based on gait analysis. The aims of this study were to explore if pelvis-thorax coordination parameters could be valuable instrument outcomes to achieve this evaluation by assessing their reliability, discriminant capacity and responsiveness.

**Methods:** Three groups of subjects; healthy, hip OA patients with severe disease (defined as indication to surgery), hip OA patients with less severe disease (no indication to surgery) were included. Hip OA patients with severe disease were evaluated before and 6 months after surgery. Subjects had to perform a gait analysis at comfortable speed, and pelvis-thorax coordination was evaluated. The correlations with clinical and structural parameters, as well as reliability, discriminant capacities and responsiveness, were assessed.

**Results:** The pelvis-thorax coordination in the coronal plane during walking was correlated to clinical and to structural severity in hip OA patients (*R*^2^ = 0.13). The coronal plane coordination allowed to discriminate healthy subjects from all hip OA patients (sensibility = 0.86; specificity = 0.59). Moreover, when comparing OA patients only, coronal plane coordination allows to discriminate patients with indication of surgery from those with no indication of surgery (sensibility = 0.72; specificity = 0.72). Moreover, the pelvis-thorax coordination demonstrated an excellent reliability and a good responsiveness.

**Conclusion:** Changes in the pelvis-thorax coordination might refer to different mechanisms, from analgesia to motor control plasticity, and might be a possible explanation for the weak correlation between structure and symptoms in hip OA patients. Moreover, such parameter might be used as an objective outcome in hip OA clinical trials.

**Clinical Trials Registration:** www.ClinicalTrials.gov, identifier: NCT02042586 and NCT01907503.

## Key Messages

- The pelvis-thorax coordination in the coronal plane during walking is correlated to the clinical and structural severity of hip OA.
- It can be used to differentiate healthy subjects, hip OA patients with severe disease, and hip OA patients with less severe disease.
- Objective Clinical Gait Analysis parameters demonstrates excellent reliability and good responsiveness.

## Introduction

Osteoarthritis is a common degenerative joint disease that is characterized by a progressive destruction of cartilage. It can affect many joints, but weight-bearing joints such as knees and hips are especially vulnerable. The functional disability induced by hip osteoarthritis (OA) has a significant impact on the patient's health-related quality of life (Zhang et al., 2008). Although several disease-specific functional questionnaires are widely used to assess functional disability (Ornetti et al., 2009), they are often subjective and reveal discrepancies between the patient's and the clinician's assessment of the disease (Lieberman et al., 1996). Therefore, it would be of interest to combine such self-assessment questionnaires with an instrument that objectively quantifies functional impairment in hip OA.

Clinical Gait Analysis is already fully incorporated into clinical decision-making for patients with complex neurological gait disorders (Baker, 2006), and it is a promising approach for the assessment of gait pattern and quantification of functional disability in patients with chronic joints diseases (Foucher et al., 2007; Ornetti et al., 2010a; Laroche et al., 2011; Longworth et al., 2018). In this sense, various gait analysis protocols have been used to report a reduction in walking speed, stride length, maximum hip flexion and extension in hip OA patients (Perron et al., 2000; Laroche et al., 2011; Martz et al., 2016; Rosenlund et al., 2016). Moreover, these changes may occur before the appearance of clinically measurable functional disability, potentially facilitating earlier and more effective medical care (Chen, 2007; Longworth et al., 2018).

Though Clinical Gait Analysis tends to focus on the identification of gait deviations at the lower limbs, several authors have emphasized the information provided by of pelvis-thorax coordination in normal and disabled gait (Lamoth et al., 2002a, 2006a). Indeed, movements between pelvis and thorax are related to the “pelvic step” term, firstly introduced by Ducroquet et al. (1965). This author proposed that the pelvis achieves a forward rotation during swing and an opposite rotation near the end of the stance phase. This rotation should thus be counterbalanced, either directly by counter-rotating the shoulder girdle, or indirectly by swinging arm (Bruijn et al., 2008). Consequently, in normal gait, the pelvis and the scapular girdle tend to move in opposite phases, resulting in a high phase shift between the two waveform segments. In some pathological cases, this mechanism could be altered, resulting in a reduced phase shift between the two waveform segments that can thus be an interesting biomarker.

On the contrary, a scapular girdle which is stable relative to the pelvic segment results in a low phase shift between the two segments, implying a more rigid gait with lower variability (Lamoth et al., 2006b). This may be of importance in hip OA, since one can expect that the severity of the disease might modify the pelvis-thorax coordination. Indeed, several hip OA-related impairments (e.g., pain, paresis, joint stiffness) may impact pelvis-thorax coordination and thus reduce the existing phase shift between the two segments (Lamoth et al., 2006a) in the coronal and transverse planes. Both coronal and transverse phase shifts have potentially different contribution either in equilibrium control or in mechanical efficiency of gait (Earhart, 2013).

To our knowledge, changes in the pelvis-thorax coordination have been documented for some disorders but not for hip OA (Lamoth et al., 2002b; Bruijn et al., 2008). The first aim of the present study was to evaluate whether the pelvis-thorax phase shift in the coronal and transverse planes is altered in hip OA patients, and, if so, whether it reflects disease severity. Our second objective was to evaluate the reliability, discriminant capacity and responsiveness of these measures in order to assess whether they are potential objective measures of hip OA disability.

## Methods

### Study Design

**Figure 1:**
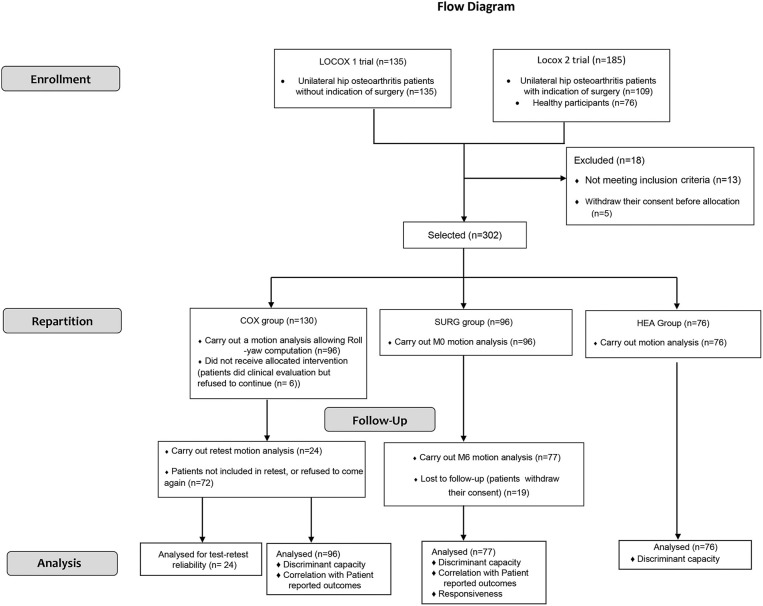
Flow diagram of the participants involved in this study.

This study is a prospective longitudinal mono-centric trial. Two separate trials (Locox 1: NCT02042586; Locox 2: NCT01907503) were used (details in Figure 1). It conforms to the COnsensus-based Standards for the selection of health Measurement INstruments (COSMIN) guidelines to perform the evolution of psychometric properties of outcome measurement instruments (Mokkink et al., 2010).

### Participants

Three categories of participants were selected: (1) HEA: healthy participants, (2) COX: unilateral symptomatic hip OA patients with no indication for total hip arthroplasty from the treating rheumatologist, and (3) SURG: unilateral symptomatic hip OA patients undergoing a total hip replacement surgery. The study presented here is ancillary of both trials presented above.

Hip OA was identified using the American College of Rheumatology Criteria (Altman et al., 1991). Exclusion criteria for hip OA subjects were OA flare, secondary hip OA, painful ankle, knee or foot disorder, acute or chronic back pain, Parkinson's disease, neural disorders, uncontrolled diabetes, cardiac or respiratory failure, inability to understand the procedures, or any major cause of inability to perform gait analyses.

Indication of surgery was based on both a Harris Hip Score (HHS) <70 and a Kellgren and Lawrence Grade 3 or 4. HHS is widely used throughout the world for evaluating outcome for THR (Harris, 1969). The indication for THR is particularly pain and impaired physical function, which are the two dominating domains in HHS (0–100, max-min). Pain receives 44 points, function 47 points, range of motion 5 points, and deformity 4 points. Function is subdivided into activities of daily living (14 points) and gait (33 points). A total score of <70 is considered a poor result; 70–80 is considered fair, 80–90 is good, and 90–100 is an excellent result (Nilsdotter and Bremander, 2011).

The protocols were developed in compliance with the Declaration of Helsinki and the Good Clinical Practice (ICH Harmonised Tripartite Guideline, 1996). They were approved by the local ethics committee (CPP Est I, Dijon, France) and all subjects signed an informed written consent form prior to inclusion.

### Outcomes

#### Evaluation of Hip OA Severity

The clinical and structural characteristics of the hip OA in COX and SURG patients were collected at inclusion, using the following measurements:

- The HOOS score (Ornetti et al., 2010b) is a validated extension of the Western Ontario and McMaster Universities Arthritis Index (WOMAC) (Bellamy et al., 1988). It is divided into five subscales covering five domains impacted by hip OA (symptoms, pain, quality of daily living, sport and recreational activities, and quality of life). Each subscale is rated 0–100 (worst to best scale).
- The Lequesne index (Lequesne et al., 1987) of severity for hip OA.
- An auto-evaluation of pain using a visual analog scale (VAS 0-10).
- Structural severity was evaluated using an anteroposterior weight-bearing radiograph of the pelvis. The radiographic grade, according to the Kellgren and Lawrence classification, was obtained by an experienced reader (Kellgren and Lawrence, 1957).

All hip OA severity parameters were evaluated by experienced rheumatologists (JFM, PO). The indication or non-indication of surgery was determined by experienced orthopedists.

#### Clinical Gait Analysis

Clinical Gait Analysis (CGA) was performed once in HEA and COX subjects except those enrolled in test-retest analyses. An additional CGA was performed in some COX subjects after a 2-week interval in order to evaluate reliability. Only this group of patients was selected to assess reliability of the proposed outcome instruments in order to avoid schedule issues related to the surgery. Finally, SURG subjects were evaluated twice: 15 days before surgery (SURG_M0_) and 6 months after surgery ± 1-month (between 5 and 7 months post-op) due to clinical schedule related delays (SURG_M6_). In all subjects evaluated twice, the same conditions (i.e., same protocol, same experimenter, and same time of day) were maintained for the two CGA sessions. All subjects were equipped with reflective cutaneous markers positioned following the conventional gait model (Davis et al., 1991). Subjects were then instructed to walk barefoot at a comfortable, self-selected speed on a 10-meter walkway (“Walk as if you were in the street”). Eight optoelectronic cameras (Vicon MX, Vicon®, Oxford, UK) sampled at 100 Hz were used. Marker trajectories were interpolated and smoothed by a 4th-order lowpass Butterworth filter with a cut-off frequency of 10 Hz. Gait cycle events (i.e., foot strike and foot off) were determined using a previously defined kinematic-based algorithm (Zeni et al., 2008). Briefly, this algorithm consists in identifying changes from positive to negative of the antero-posterior velocity vector of a heel marker to detect foot strikes, and changes from negative to positive of the antero-posterior velocity vector of a toe marker to detect foot offs. Both markers are expressed in the pelvis coordinate system in this approach.

#### Assessment of Phase Shifts

Based on the CGA measurements, walking speed (m.s^−1^), transverse and coronal planes phase shifts (%) were our main variables of interest.

During walking, pelvis, and thorax segments rotate in opposite directions, yielding opposing sinusoidal waveforms, transverse and coronal planes phase shifts illustrate the lag between these waveforms (i.e., the pelvis-thorax coordination). The less the movements of the segments are synchronized (i.e., move in opposite directions), the more these phase shifts increase.

The phase shifts between the pelvic and thorax rotations were calculated using the continuous relative phase method using a technique described by van Emmerik and Wagenaar (1996). This method has been widely used for quantifying the coordination between different oscillating body segments, including pelvis-thorax coordination (Mangone et al., 2011; Seay et al., 2011). It can be defined as the difference between the respective phase angle of each segment (Lamoth et al., 2002a). Finally, as the mean phase shift is a measure based on an angular scale from 0 to 360°, the mean value for each participant was calculated using a circular mean (Berens, 2009). 180° was considered as a complete unphase and 0° (360°) as a complete phase. All results were then reported between 0 and 50% (i.e., 0% being a complete phase and 50% a complete unphase).

### Statistical Analyses

Statistical analyses were done using Statistica v10.0 (StatSoft, Tulsa, USA) and Matlab with statistical toolbox (Matlab 2015b, The MathWorks, Natick, USA). Statistical significance was set at *P* < 0.05.

We hypothesized that the pelvis-thorax phase shift could be a valuable discriminant tool if it allowed us to accurately differentiate COX and SURG patients from HEA individuals, i.e., discriminant ability should be around 0.8 to be used in clinical practice. The sample of the selected participants allowed to achieve a 79% power to detect a difference of 0.1 between the area under the ROC curve (AUC) of 0.8. Differences will be assessed under the alternative hypothesis (corresponding to the target discriminant ability of the phase shift) and under the null hypothesis (discriminant ability as low as 0.7) using a two-sided z-test at a significance level of 0.05.

#### Step 1. Evaluation of Reliability, Discriminant Capacity, and Responsiveness

##### Step 1a—Reliability in hip OA

The measurements obtained on COX patients who agreed to perform two CGA were used. The intraclass correlation coefficient (ICC_(2,k)_) and the 95% confidence interval (CI) were computed to evaluate measurement agreements. An ICC higher than 0.7 was considered as good, and higher than 0.9 as excellent (Weir, 2005). The magnitude of the difference between the two sessions was assessed by the Hedges g and the 95% CI. The magnitude of the difference was considered small (0.2 < g ≤ 0.5), moderate (0.5 < g ≤ 0.8), or large (g > 0.8) (Daly and Cohen, 1978).

Bland and Altman plots were used to show the agreement and correlation between the two sessions (Bland and Altman, 1986). The observed difference in phase shifts between the two sessions was plotted against the mean result of each subject.

##### Step 1b—Discriminant capacity

A one-way analysis of variance (ANOVA) was conducted to detect potential differences in the transverse and coronal planes phase shifts in the three groups (HEA, COX, SURG_M0_). If the ANOVA revealed a difference, a Tukey's *post hoc* test for intergroup comparisons was then conducted. Mean value and standard deviation are provided.

The capacity of the pelvis-thorax phase shifts to discriminate hip OA patients (COX and SURG_M0_) from HEA participants, and surgical (SURG_M0_) from non-surgical (COX) hip OA patients, was also evaluated using the Receiver Operation Characteristic (ROC). The area under the curve (AUC) and the 95% CI was quantified, as well as the optimum sensitivity/specificity ratio [Youden Index method (Youden, 1950)].

##### Step 1.c—Responsiveness in hip OA

The analysis conducted in Step 1b also included the SURG_M6_ group. The responsiveness of the test was thus assessed by detecting potential differences in the transverse and coronal planes phase shifts between SURG_M0_ and SURG_M6_. To this end, the standardized response mean (SRM), the mean change between groups divided by the standard deviation of the mean change, and the effect size (ES), the mean score change between groups divided by the standard deviation of pre-surgery values, were calculated.

#### Step 2—Do the Pelvis-Thorax Phase Shifts Reflect the Severity of Hip OA?—Correlation With Patient-Reported Outcomes

Correlations between the transverse and coronal planes phase shifts and both clinical and structural parameters (i.e., HOOS domains, Lequesne's index, VAS pain, Kellgren and Lawrence grading scale) were sought out in all included hip OA patients (COX and SURG_M0_) using either univariate Pearson correlation coefficients or the linearity of the ANOVA. All variables significantly correlated with the transverse and coronal planes phase shifts in the univariate analysis were included in a multiple regression analysis. It was adjusted for walking speed, which is known to greatly affect gait kinematics (Schwartz et al., 2008), and particularly the transverse and coronal planes phase shifts (Lamoth et al., 2006a). The same analysis was performed for the coronal plane phase shift. We also computed Variation Inflation Factor between explicative variables and considered a value higher than 10 to admit collinearity between variables (Kutner et al., 2004).

The correlations were considered very low [0.15 < *r* < 0.24], low [0.25 < *r* < 0.49], moderate [0.50 < *r* < 0.69], high [0.70 < *r* < 0.89], or very high [0.90 < *r* < 1.00] according to Munro's correlation descriptors (Munro, 1997).

## Results

### Participants

The study course is described in the flow diagram (Figure 1). In total, 76 HEA, 130 COX and 96 SURG subjects were recruited. 20 SURG subjects did not complete CGA or were lost to follow-up after surgery, respectively, and were thus excluded from the analysis. Twenty-four COX subjects agreed to perform a second CGA.

The demographic characteristics and the clinical and structural data obtained at baseline for all subjects are provided in Table 1. The Lesquesne index was only collected in the COX group. Before surgery, SURG subjects were significantly more aged than COX group. SURG subjects also have higher significantly BMI then COX. Hence, SURG subjects have significant lower functional outcomes (HOOS Score, EVA pain) than COX. 6 months after surgery, results are strictly opposite (except for age and BMI).

**Table 1:** Characteristics of the participants (HEA: healthy participants, COX: unilateral symptomatic hip osteoarthritis patients with no indication for surgery; SURG: unilateral symptomatic hip osteoarthritis patients undergoing a total hip replacement; M0: 15 days prior to surgery; M6: 6th months after surgery).

	**HEA**	**COX**	**SURG**	
			**M0**	**M6**
Sex [Total (M/F)]	34/42	38/58	35/42	
Age (mean years, SD)	58.1 (15.5)	61.1 (8.2)	67.1 (9.9)^*^	
Body Mass Index (mean Kg/m2, SD)	24.9 (3.7)	26.2 (3.9)	28.6 (5.4)^*^	
Kellgren and Lawrence grade (number of patients)				
I		0	2	
II		46	12	
III		36	32	
IV		4	30	
Lequesne's score (mean score, SD)		8.3 (3.5)		
HOOS Symptoms (mean score SD)		55.7 (18.1)	42.8 (18.5)^*^	81.8 (16.8)^*^
HOOS Pain (mean score, SD)		55.0 (19.0)	42.8 (18.3)^*^	84.4 (18.8)^*^
HOOS Function (mean score, SD)		55.6 (20.7)	37.4 (16.4)^*^	81.4 (17.0)^*^
HOOS Sports and recreational activities (mean score, SD)		39.3 (24.7)	24.0 (20.1)^*^	73.6 (26.3)^*^
HOOS Quality of Life (mean score, SD)		43.7 (24.2)	27.4 (21.5)^*^	80.8 (20.7)^*^
Pain (VAS 0-10) (mean score, SD)		4.8 (2.2)	6.9 (2.0)^*^	1.4 (2.0)^*^

* *Indicate significant difference (P <0.05) between SURG and COX groups*.

#### Step 1—Correlation to Gold Standard Outcomes

##### Step 1a—Concurrent validity. Correlation with patients reported outcomes

On univariate analysis, the transverse and coronal planes phase shifts were correlated to HOOS pain, HOOS function, VAS-pain, Lequesne index and Kellgren & Lawrence grade (Table 2). Values adjusted for walking speed are provided in Table 3. All parameters were correlated to walking speed, except the Kellgren & Lawrence grade. The coronal plane phase shift was correlated to HOOS Function, HOOS Quality of Life, VAS-pain and Kellgren & Lawrence grade, while the transverse plane phase was only correlated to Kellgren & Lawrence grade. Variation inflation Factor was below 5 for all the explicative variables.

**Table 2:** Pearson Correlation Coefficients between transverse and coronal planes phase shifts (pelvis-thorax coordination) during walking, and clinical and structural parameters for (i) all hip osteoarthritis participants at M0 (COX: unilateral symptomatic hip osteoarthritis patients with no indication to surgery, *n* = 96; and SURG_M0_: unilateral symptomatic hip osteoarthritis patients undergoing a total hip replacement at M0: 15 days prior to surgery, *n* = 77) and (ii) SURG_M6_ (unilateral symptomatic hip osteoarthritis patients undergoing a total hip replacement at M6: 6th months after surgery, *n* = 77).

**Parameters**		**Pearson Correlation Coefficient**		
		***n***	**Transverse plane phase shift**	**Coronal plane phase shift**
COX + SURG_M0_	Lequesne (only SURG_M0_)	96	0.06	−0.26^*^
	HOOS Symptoms	173	0.14	0.17^*^
	HOOS Pain	173	0.17^*^	0.26^*^
	HOOS Function	173	0.17^*^	0.32^*^
	HOOS Activities	173	0.13	0.23^*^
	HOOS QoL	173	0.12	0.24^*^
	Pain (VAS 0-10)	173	−0.16^*^	−0.27^*^
	Kellgren and Lawrence grade	173	−0.25^*^	−0.34^*^
SURG_M6_	HOOS Symptoms	77	0.05	−0.16
	HOOS Pain	77	−0.13	−0.14
	HOOS Function	77	0.02	−0.10
	HOOS Activities	77	>0.01	0.02
	HOOS QoL	77	−0.08	0.01
	Pain (VAS 0-10)	77	0.02	0.05

* *Indicates significant relationship between parameters in univariate analysis*.

**Table 3:** Multiple regression between the transverse and coronal planes phase shifts (pelvis-thorax coordination) during walking and clinical and structural parameters, adjusted for gait velocity for both COX (unilateral symptomatic hip osteoarthritis patients with no indication for surgery) and SURG_M0_ (unilateral symptomatic hip osteoarthritis patients undergoing a total hip replacement; M0: 15 days prior to surgery).

**Parameters**		**Multiple regression (*****p*****-value)**			
		**Transverse plane phase shift**	**Coronal plane phase shift**	**Walking velocity**	**Multiple *R*^2^**
COX + SURG_M0_	Lequesne (only SURG_M0_)	−	0.20	**<0.001**	0.22^*^
	HOOS Symptoms	−	0.39	**0.01**	0.06^*^
	HOOS Pain	0.89	0.07	**<0.001**	0.11^*^
	HOOS Function	0.46	**0.01**	**0.001**	0.16^*^
	HOOS Sports and recreational activities	−	0.11	**0.005**	0.09^*^
	HOOS Quality of life	−	**0.04**	**0.03**	0.08^*^
	Pain (VAS 0-10)	0.57	**0.04**	**0.003**	0.13^*^
	Kellgren and Lawrence grade	**0.05**	**<0.001**	0.39	0.13^*^

*The dash indicates that there was no significant correlation between parameters in univariate analysis. Significant p-values in the multiple regression analyses are bolded*.

* *Indicates significant relationship*.

#### Step 2—Discriminant Capacity

**Figure 2:**
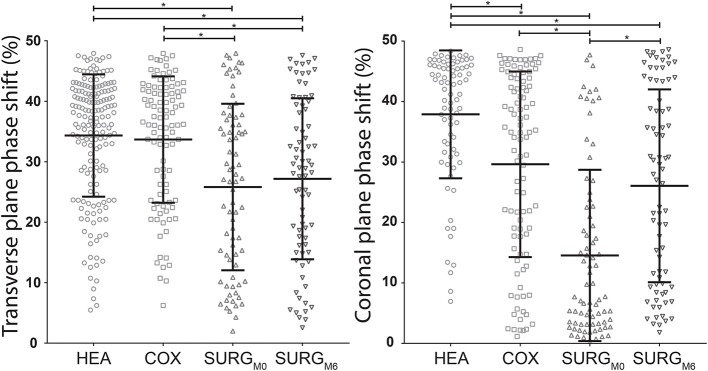
Distribution with mean (horizontal middle bar) and standard deviation (horizontal upper and lower bars) of the transverse and coronal planes phase shifts (pelvis-thorax coordination) during walking in the HEA, COX, SURG_M0_, and SURG_M6_ subjects. Significant differences (ANOVA and Tukey) are indicated with a * (HEA: healthy participants, COX: unilateral symptomatic hip osteoarthritis patients with no indication to surgery; SURG: unilateral symptomatic hip osteoarthritis patients undergoing a total hip replacement; M0: 15 days prior to surgery; M6: 6th month after surgery).

As indicated in Figure 2, for the transverse plane phase shift, the HEA and COX groups showed a significantly higher desynchronization than the SURG_M0_ group, while there was no difference between HEA and COX groups.

The results for the coronal plane phase shift were different since this calculation allowed to discriminate between all groups (Figure 2). Moreover, the discrimination between COX and SURG_M0_ tended to be more pronounced.

**Figure 3:**
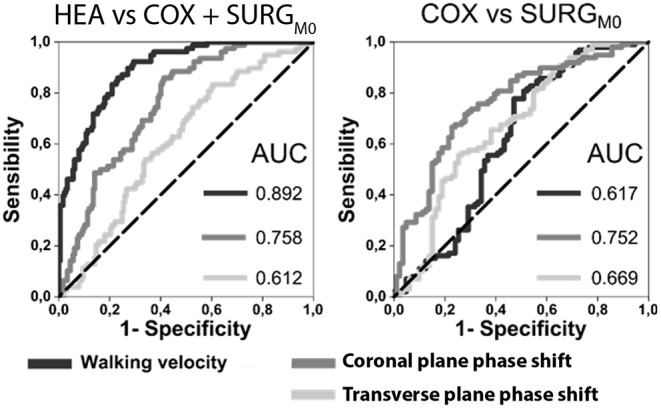
ROC curves for walking velocity, transverse and coronal planes phase shift for HEA vs. HOA and COX vs. SURG_M0_ with the Area Under the Curves (AUC) indicated (HEA: healthy participants, COX: unilateral symptomatic hip osteoarthritis patients with no indication to surgery; SURG: unilateral symptomatic hip osteoarthritis patients undergoing a total hip replacement; M0: 15 days prior to surgery; M6: 6th month after surgery).

Table 4 and Figure 3 show the results of the ROC analysis. Walking speed (AUC = 0.89) and the coronal plane phase shift (AUC = 0.76) were the parameters that differentiated hip OA patients from healthy participants most accurately.

**Table 4:** Results of the Receiver Operation Characteristics (HEA vs. COX+SURG_M0_ and COX vs. SURG_M0_): area under the curve (AUC), sensitivity and specificity parameters of the transverse, coronal planes phase shift (pelvis-thorax coordination) and the walking velocity as well as the respective threshold value are presented (HEA: healthy participants, COX: unilateral symptomatic hip osteoarthritis patients with no indication for surgery; SURG: unilateral symptomatic hip osteoarthritis patients undergoing a total hip replacement; M0: 15 days prior to surgery; M6: 6th months after surgery).

**Comparison**	**Parameters**	**AUC** **(95% CI)**	**Sensitivity/specificity**	**Threshold value**
HEA vs. COX+SURG_M0_ patients	Transverse plane phase Shift (% gait cycle)	0.61 (0.54–0.68)	0.83/0.40	28.0
	Coronal plane phase shift (% gait cycle)	0.76 (0.70–0.82)	0.86/0.59	27.5
	Walking velocity (m.s^−1^)	0.89 (0.85–0.93)	0.92/0.72	1.07
COX vs. SURG_M0_	Transverse plane phase Shift (% gait cycle)	0.68 (0.59–0.76)	0.56/0.74	36.2
	Coronal plane phase Shift (% gait cycle)	0.78 (0.71–0.85)	0.72/0.72	19.6
	Walking velocity (m.s^−1^)	0.63 (0.54–0.72)	0.78/0.53	0.88

When considering only hip OA patients, the most accurate parameters for discriminating patients according to severity (surgery vs. non-surgery patients) were the coronal plane phase shift (AUC = 0.78) and the transverse plane phase shift (AUC = 0.68).

##### Step 2.a — Reliability

The test-retest reliability of the phase shifts observed in the 24 COX subjects is provided in Table 5. The reliability of both the transverse and coronal planes phase shifts was excellent with ICC > 0.9, and Hedges g lower than 0.2, indicating a small magnitude of change.

**Table 5:** Reliability of the evaluation of the transverse and coronal plane phase shifts (pelvis-thorax coordination) during walking.

**Parameters**	**Transverse plane phase shift**	**Coronal plane phase shift**
Number of patients	24	24
Mean Session 1: % of gait cycle (SD)	35.1 (9.5)	32.7 (13.2)
Mean Session 2: % of gait cycle (SD)	34.3 (12.0)	31.9 (14.5)
ICC_(2, k)_ (95%CI)	0.95 (0.89–0.98)	0.91 (0.80–0.96)
Hedges' g (95% CI)	0.07 (−0.48–0.63)	0.06 (−0.50–0.61)

*24 COX subjects were evaluated twice. One evaluation at the inclusion in the trial (Session 1) and one after a two-week interval (Session 2)*.

**Figure 4:**
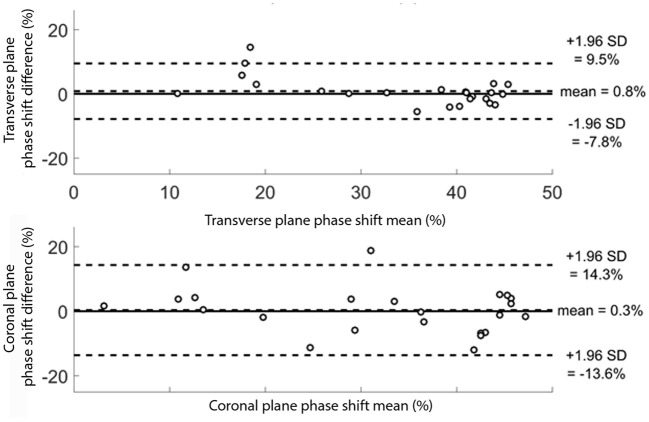
Bland Altman plots for the 24 subjects for the transverse and coronal planes phase shift (pelvis-thorax coordination) during walking. The subjects were evaluated twice. For each subject, the mean result of the two evaluations is plotted against the difference between the two evaluations. The transverse and coronal planes phase shift results are given in percentage.

The mean differences between the two sessions were 0.8% (95% CI [−7.8; 9.5]) and 0.3% (95% CI [−13.6; 14.3]) for the transverse and coronal planes shift phases, respectively, as indicated on Bland-Altman plots (Figure 4). These plots also highlight that there was no clear relation between the mean and the difference of the two sessions.

##### Step 2.b—Responsiveness

There was no significant difference between SURG_M0_ and SURG_M6_ for the transverse plane phase shift (Figure 2). On the contrary, the coronal plane phase shift was increased significantly 6 months after surgery, reaching values similar to those obtained in patients with less severe hip OA (Figure 2). Details are presented in Table 6.

**Table 6:** Responsiveness of the transverse and coronal planes phase shifts (pelvis-thorax coordination) during walking.

	**Pre-surgery value** **(SD)**	**6-month** **post-surgery** **value (SD)**	**Mean change** **(SD)**	**Effect size**	**Standardized** **response mean**
Transverse plane phase shift (% gait cycle)	25.8 (13.7)	27.2 (13.2)	1.4 (9.6)	0.10	0.14
Coronal plane phase cycle (% gait cycle)	14.5 (14.1)	26.1 (15.4)^*^	11.5 (15.4)	0.82	0.75

*Pre-surgery, 6-month post-surgery, mean change and their standard deviation (SD), effect size and standardized response mean are provided for the patients of the SURG group (unilateral symptomatic hip osteoarthritis patients undergoing a total hip replacement)*.

* *Indicates significant difference between pre and post-surgery*.

The effect size and SRM of the transverse plane phase shift were 0.10 and 0.14, respectively (i.e., small effect). The effect size and SRM of the coronal plane phase shift were 0.82 and 0.75, respectively (i.e., large effect).

## Discussion

To our knowledge, the present study is the first to provide information regarding pelvis-thorax coordination in hip OA patients, though this type of quantification has been used extensively in patients suffering from low back pain (Lamoth et al., 2006b; Huang et al., 2011; Seay et al., 2014). The present results suggest that the coordination between the pelvis and thorax rotations in the coronal plane during walking is correlated to the clinical and structural severity of hip OA, and can be used to differentiate healthy subjects, hip OA patients with severe disease (defined as indication for joint replacement), and hip OA patients with less severe disease (defined as no indication for joint replacement). Moreover, this objective CGA parameter demonstrates excellent reliability and good responsiveness.

These results must be interpreted carefully, since this work has several potential limitations. First, we did not evaluate the reliability of phase shift coordination in SURG group, i.e., the group of more severe hip OA with an indication for total joint replacement; this can potentially limit the generalizability of our results in hip OA patients and needs to be assessed in further studies. Secondly, we did not evaluate the somatosensory aspects of the adaptation of pelvis-thorax coordination. Coronal and transverse plane might be linked to different central control processes. Thus, further studies should link kinesthetic sense and muscle strength measurements to the transverse and coronal planes phase parameters, in order to better explore patient adaptations. Thirdly, the type of limping was not assessed since patients may present Trendelenburg or avoidance strategies that impact undoubtedly their pelvis-thorax coordination. Such dichotomy needs to be done in further studies to determine if the limping strategy affects differentially the pelvis-thorax coordination during gait. Finally, the age and BMI of the patients with more severe disease were higher.

To our knowledge, the present study reports a relationship between a structural and a gait parameter in hip OA for the first time. This is of particular interest since the relationship between clinical and structural parameters remains controversial (Gossec et al., 2009; Chu Miow Lin et al., 2011; Foucher and Wimmer, 2012; Kumar et al., 2015, 2018). One possible explanation for the weak correlation between structure and cross-sectional symptoms might be differences among hip OA patients in gait adaptation to reduce pain. Nevertheless, structural integrity of the hip was generally not related to the functional capacity of the patients (Gossec et al., 2007). Moreover, these parameters were also notably different and the correlation, even weak, between domains of different natures should encourage further studies to evaluate this hypothesis before proposing it in a clinical setting.

Currently, the evaluation of functional capacity in hip OA patients is mostly based on patient-reported outcome instruments. The advantages of such instruments are that they evaluate function from the patients' point of view and that some have been validated in studies which demonstrated their good psychometric properties (Nilsdotter and Bremander, 2011). However, these instruments are subjective and highlight discrepancies in patients' and clinicians' evaluation of the disease (Lieberman et al., 1996). Therefore, it would be of interest to combine self-assessment questionnaires with an instrument that objectively quantifies functional impairment in hip OA, such as gait analysis. To assess a potential outcome measure, it is necessary to evaluate its psychometric properties, as defined by the OMERACT filter (Boers et al., 1998). The OMERACT filter checks that a potential outcome measure is (a) feasible, (b) truthful, i.e., reflects what it is supposed to reflect (validity), and (c) discriminant, which includes sensitivity to change. The present results suggest that the evaluation of the coordination of the pelvic and thorax segments in the coronal plane during walking might be used a potential tool for assisting clinician in the evaluation of hip OA patients, and as an objective measurement in clinical trials. On the other hand, the main problem with the use of gait analysis is feasibility: such analyses are more expensive and time consuming than collecting patient reported outcomes. However, it can be of interest to propose a new reduced set of gait markers, such as the coronal plane phase shift evaluation, in order to make gait analysis easier, more feasible, and less costly. Therefore, further studies are needed to better evaluate the psychometric properties of this potential outcome measure.

In hip OA patients, the present results suggest that the coronal plane phase shift was correlated to clinical severity and stiffness. A first assumption could be made in relation with the so-called “balance of Pauwels.” During single leg stance, the lever arm of the body center of mass being higher than the lever arm of the abductor muscles, a high hip joint compressive force is induced by these muscles to control the drop of the contralateral pelvis, with an amplitude of three to four times the body weight (Pauwels, 1976; Neumann, 1989; Sims, 1999). In order to decrease this compressive force and the related pain, a compensation could be to reduce the lever arm of the body center of mass, for example by desynchronizing the pelvis and the thorax. By leaning their pelvis and thorax toward the side of the painful hip, the constraints on the center of the hip joint should be reduced (Murray et al., 1971; Neumann, 1989). Both clinical parameters and coronal plane phase shift were improved at 6 months post-surgery compared to pre-surgery, which might be in favor of this assumption. However, the coronal plane phase shift returned to the levels observed in non-surgical hip OA patients, while post-surgery patients were less symptomatic than non-surgical patients. This assumption remains thus unclear and might be related to the abductor strength. Indeed, several studies has showed that OA patients have limited abductor strength (Marshall et al., 2016). Further studies might thus be needed to evaluate the relationship between pain, abductors strength and pelvis-thorax coordination.

On the contrary, the transverse plane phase shift was related to structure but not to clinical parameters, and it was different only in patients with severe hip OA. Moreover, it was not responsive 6 months after surgery. The transverse plane phase coordination might be more centrally dependent as suggested in low back patients (Lamoth et al., 2006b). During locomotion, the coordination and consistency of thorax movements form the foundations of postural control (Earhart, 2013). In addition, ability to regulate posture in the case of impaired mechanics is a challenging but key process to guarantee successful coordination (Earhart, 2013). Moreover, the pain adaption model first formulated by (Lund et al., 1991), may partially explained this phenomenon (Lund et al., 1991; Arendt-Nielsen et al., 1996). This regulation implies, in hip OA patients, adaptation of the nervous system by modulation of muscles activities from the beginning of the disease. Because this neural plasticity may not be reversed immediately, we can expect the mid-term evaluation done in the present study was too early to reveal all the adjustments to the gait pattern. However, this hypothesis should be confirmed with longer follow-up in order to show the time needed for transverse plane phase coordination to return to the control values.

Thus, in hip OA patients, the changes observed on pelvis-thorax coordination may be linked to different mechanisms altering normal gait. However, in a common clinical setting, this coordination may not be readily measurable, which limits the practical significance of this outcome. Arguably, the overall pattern of coordination, as described by the relative phase between pelvis and thorax segments, is related to the level of the OA severity of the patients. More broadly, the overall pattern of coordination is closely related to the level at which the clinician evaluates the integrity of movement patterns (Lamoth et al., 2002b). Thus, the quantification of the pelvis-thorax coordination may be a valuable additional argument helping to establish and support the clinical diagnostic.

## Data Availability Statement

The datasets generated for this study are available on request to the corresponding author.

## Ethics Statement

The studies involving human participants were reviewed and approved by CPP Est I, Dijon, France. The patients/participants provided their written informed consent to participate in this study.

## Author Contributions

FM: analysis and interpretation of data, critical revision of the article for important intellectual content, analysis and interpretation of data. AN: drafting of the article, analysis and interpretation of data, statistical expertise, and collection and assembly of data. PO: conception and design, obtaining of funding, provision of study materials or patients analysis and interpretation of data, and critical revision of the article for important intellectual content. AB: analysis and interpretation of data and statistical expertise. CB: revising the article critically for important intellectual content, and final approval of the version to be submitted. CM: conception and design of the study and obtaining of funding. AG: revising the article critically for important intellectual content and final approval of the version to be submitted. J-FM: conception and design, obtaining of funding, provision of study materials or patients analysis and interpretation of data, critical revision of the article for important intellectual content and statistical expertise. DL: conception and design, obtaining of funding, analysis and interpretation of data, critical revision of the article for important intellectual content, statistical expertise, and collection and assembly of data.

### Conflict of Interest

The authors declare that the research was conducted in the absence of any commercial or financial relationships that could be construed as a potential conflict of interest.
